# Delirium risk stratification in consecutive unselected admissions to acute medicine: validation of externally derived risk scores

**DOI:** 10.1093/ageing/afv177

**Published:** 2016-01-13

**Authors:** Sarah T. Pendlebury, Nicola Lovett, Sarah C. Smith, Emily Cornish, Ziyah Mehta, Peter M. Rothwell

**Affiliations:** 1 NIHR Oxford Biomedical Research Centre, John Radcliffe Hospital, Oxford, UK; 2 Departments of General (Internal) Medicine and Geratology, John Radcliffe hospital, Oxford, UK; 3 Stroke Prevention Research Unit, Nuffield Department of Clinical Neurosciences, John Radcliffe Hospital and the University of Oxford, Oxford OX3 9DU, UK

**Keywords:** delirium, prediction, risk stratification, risk scores, acute medicine

## Abstract

**Background:** reliable delirium risk stratification will aid recognition, anticipation and prevention and will facilitate targeting of resources in clinical practice as well as identification of at-risk patients for research. Delirium risk scores have been derived for acute medicine, but none has been prospectively validated in external cohorts. We therefore aimed to determine the reliability of externally derived risk scores in a consecutive cohort of older acute medicine patients.

**Methods:** consecutive patients aged ≥65 over two 8-week periods (2010, 2012) were screened prospectively for delirium using the Confusion Assessment Method (CAM), and delirium was diagnosed using the DSM IV criteria. The reliability of existing delirium risk scores derived in acute medicine cohorts and simplified for use in routine clinical practice (USA, *n* = 2; Spain, *n* = 1; Indonesia, *n* = 1) was determined by the area under the receiver operating characteristic curve (AUC). Delirium was defined as prevalent (on admission), incident (occurring during admission) and any (prevalent + incident) delirium.

**Results:** among 308 consecutive patients aged ≥65 (mean age/SD = 81/8 years, 164 (54%) female), existing delirium risk scores had AUCs for delirium similar to those reported in their original internal validations ranging from 0.69 to 0.76 for any delirium and 0.73 to 0.83 for incident delirium. All scores performed better than chance but no one score was clearly superior.

**Conclusions:** externally derived delirium risk scores performed well in our independent acute medicine population with reliability unaffected by simplification and might therefore facilitate targeting of multicomponent interventions in routine clinical practice.

## Introduction

Delirium is an acute and fluctuating confusional state associated with increased care needs and poor outcomes [1]. Reliable delirium risk stratification will aid screening, anticipation and prevention, and enable better targeting of clinical resources [1]. Major risk factors (besides older age) include cognitive impairment, hip fracture and severe illness, but many other factors are implicated making risk prediction in individual patients difficult [1]. Formal risk scores may help but must be simple to use, have clinical credibility and be externally validated on representative cohorts [2–4].

Existing delirium risk scores derived in acute medicine generally incorporate measures of impairment (sensory, cognitive and or functional) and illness severity and or infection, but there are few validations in external cohorts and some include complex measures making them difficult to use in practice [5–10]. No studies have examined whether scores derived to predict incident delirium (occurring *de novo* during admission) will also identify any delirium (prevalent and incident delirium) and vice versa despite the fact that such a score would have clinical utility in both screening/recognition and prediction of delirium.

We therefore determined the reliability of existing acute medicine risk scores described in the literature [5–9] for any, incident and prevalent delirium in a consecutive cohort of older acute medicine patients. We also assessed the robustness of existing scores to simplification for use with data acquired by the medical team as part of routine clinical practice.

## Methods

### Patient cohort

The Oxford University Hospitals Trust (OUHT) provides services for all acute medicine patients in a population of ∼500,000 and runs an unselected medical admissions system, with the majority of patients remaining under the admitting team. In a prospective observational audit, consecutive admissions to a single team over two 8-week periods (September–November 2010 and April–June 2012) were screened for delirium on arrival and daily thereafter by the admitting team until discharge, transfer or death. The audit was undertaken to inform future service development and was approved by the Divisional Management and registered with the OUHT Audit Team. All data were routinely acquired as part of standard patient care. Some data on age-specific delirium rates, associates and outcomes from this cohort have been published previously [11].

All patients were seen within 24 h of admission by an experienced Consultant Physician (dually accredited in acute general (internal) medicine and geriatrics (S.T.P., S.C.S.)) responsible for the patient's care and at least every other day thereafter. All patients aged ≥65 had the Confusion Assessment Method (CAM) [12] and a cognitive test: Cohort 1 (2010) had the mini-mental state examination (MMSE) [13] and Cohort 2 (2012) had the abbreviated mental test score (AMTS) [14]. The cognitive test and CAM formed part of the standard OUHT clerking proforma administered by junior doctors on the STP/SCS admitting team all of whom were trained in their use as part of standard OUHT practice led by STP. Cognitive impairment was defined as AMTS < 9 or MMSE < 24 according to published cut-offs [15, 16] and/or prior diagnosis of dementia. Delirium diagnosis was made according to DSM IV criteria [17] by the responsible physician (S.T.P., S.C.S.) after discussion with the rest of the medical team and was categorised as any delirium (occurring at any point during admission), prevalent delirium (on admission or within the first 48 h) or incident delirium (occurring after the first 48 h). If delirium was present on admission, a 48-h period without evidence of delirium was required before a new episode of delirium occurring during admission could be recorded.

Demographic data, presenting complaint and potential risk factors were recorded from the patient, relatives and primary care physician (GP) and medical records including living arrangements (care home versus home with care package versus home without formal care) and clinical and physiological parameters (see below). Prior diagnosis of dementia was recorded if the diagnosis was present in the GP letter, reported by the patient or relative or had been recorded previously in the patient's notes. Vision and hearing impairment was recorded if noted in the medical history or was evident during patient admission or subsequent interview. Admission physiological parameters (pulse, temperature and respiratory rate) were taken from the patient's chart. Systemic inflammatory response syndrome (SIRS) was used as a measure of illness severity since it required only routinely collected clinical data and was classed as positive if two or more of the following were present: heart rate >90 bpm, temperature <36 or >38°C, respiratory rate >20 breaths per minute, white blood cell count <4 × 10^9^ or >12 × 10^9^ cells per litre [18].

### Selection and adaptation of externally derived delirium risk scores

We selected delirium risk prediction scores for testing in our sample if they were derived on acute medicine cohorts [5–9] and did not examine scores derived in other environments including surgical cohorts, intensive care, the emergency department or wards restricted to frail, dependent older patients [19–21]. Existing risk scores were adapted and simplified where necessary to allow use with data acquired as part of the medical team's routine clinical assessment (Table 1). We were not able to examine the score developed by Carrasco *et al.* [10] in an acute medicine cohort since this could not be simplified for use with our dataset owing to the need for a numeric value for the Barthel index. Specifically, for all included scores, severe illness was defined by SIRS ≥ 2. Cognitive impairment was defined as a diagnosis of dementia and/or cognitive score below cut-off (MMSE < 24, AMTS < 9). Similarly, in the AWOL (Age, failure to spell ‘World’ backward, disOrientation to place and iLlness severity) score [8], spelling WORLD backwards and disorientation (1 point each) was replaced by a diagnosis of dementia and/or cognitive score below cut-off (MMSE < 24, AMTS < 9, 2 points). Functional dependency was defined as residence in a care home or at home with carers. In the Indonesian score [7], ‘infection with sepsis’ was defined as infection together with SIRS ≥ 2.

**Table 1.** Delirium risk scores for acute medicine used in the current study showing original development and internal validation cohort characteristics and factors used in the models

Authors	Score	Study location	Study type	Patient characteristics	Factors in the model
Inouye *et al.* Yale-Newhaven, USA [5]	Score/4 for incident delirium	6 General medicine floors. 1988–89 *n* = 107 (derivation) *n* = 174 (validation)	Prospective incident delirium, prevalent delirium excluded	Age >70 years, mean age = 79.6 + 6.6 years, 54% female. Severe dementia excluded. Numbers developing delirium *n* = 27 (derivation cohort) and *n* = 29 (validation cohort)	Vision impairment = 1 Severe illness (APACHE II + nurse assessment) = 1^a^ Cognitive impairment (MMSE < 24) = 1 Dehydration (High BUN/Cr ratio) = 1
Martinez *et al.* San Sebastian, Spain [6]	Score/3 for delirium at any point during admission (any delirium)	4 Internal medicine wards, 2008–09 *n* = 397 (derivation) *n* = 302 (validation)	Retrospective any delirium. Delirium diagnosis made on the basis of chart review at discharge. Cognition not assessed	Mean age = 76.4 ± 13.3 years, 52% female. Numbers developing delirium *n* = 76 (derivation cohort) and *n* = 52 (validation cohort)	Age ≥85 years = 1 Functional dependency = 1^b^ Psychotropic medication ≥2 drugs (antipsychotics, benzodiazepines, antidepressant, anticonvulsant, anti-dementia medication) = 1^c^
Isfandiaty *et al.* Jakarta, Indonesia [7]	Score/7 for incident delirium	Internal medicine and acute geriatrics admissions ward, 2008–10. Data extracted from records in 2011. *n* = 457	Retrospective prevalent delirium excluded. Delirium <14 days after admission diagnosed by chart review	Age ≥60 years, mean age = 69.6 ± 7.1 years, 52.5% male. Numbers developing delirium *n* = 86	Cognitive impairment = 3 Functional dependency = 2 Infection ‘without sepsis’ = 1 Infection ‘with sepsis’ = 2
Douglas *et al.* San Francisco, USA [8]	AWOL Score/4 for incident delirium	Medicine, cardiology, neurology 2010–12 *n* = 209 (derivation) *n* = 165 (validation)	Prospective incident delirium, prevalent delirium excluded	Mean age = 68.08 ± 11.96, 54% male Numbers developing delirium were *n* = 25 (derivation cohort) and *n* = 14 (validation cohort)	Age ≥80 years = 1 Failure to spell WORLD backwards = 1^c^ Disorientation to place = 1^c^ Illness severity (nurse assessment) = 1

APACHE II, Acute Physiology and Chronic Health Evaluation II score; BUN, blood urea nitrogen.

^a^Severe illness was defined by SIRS ≥ 2 in the current study rather than the APACHE II score since the latter requires arterial blood gas sampling.

^b^Assessed by the researchers using performance in six activities of daily living.

^c^Replaced by diagnosis of dementia or cognitive score below cut-off in the current study.

### Statistical analyses

We determined whether the existing acute medicine delirium risk scores could reliably identify those patients with delirium in our cohort. All scores were examined for prediction of any, prevalent and incident delirium even if originally developed specifically to predict risk of incident delirium using the areas under the receiver operating characteristic curve (AUC). To determine the performance of the scores for identifying risk of incident delirium, patients with prevalent delirium were excluded from the analyses. For analyses of prevalent delirium, all patients were included. Missing data were not imputed except for cognitive data where AUCs were calculated both without and with imputed data with missing scores imputed as normal. Statistical differences between the AUCs obtained for the existing risk scores were tested with pairwise comparisons using the *z* test. Sensitivity, specificity and positive and negative predictive values were calculated.

### Sample size calculation

Using an estimate of 33% overall delirium rate in admissions to acute general medicine aged ≥65 from previous pilot work and published estimates [1, 22–24], we calculated that a sample size of 300 would yield 100 delirium outcomes giving sufficient power to examine the reliability of the five delirium risk scores all with 3–5 risk factors (given requirement for 20 outcome events per factor examined) [25]. Although this sample size would not give the statistical power to reliably determine small differences between the different risk scores, it would allow us to determine whether individual risk scores perform better than chance (where the lower CI for the AUC is >0.5, the null hypothesis is disproved). The sample size calculation was done on the basis of detection of any delirium. We expected lower rates of incident delirium and thus less power to determine whether scores were reliable specifically for incident delirium.

## Results

Among 308 consecutive patients aged ≥65 (mean/SD age 81/8 years, 164 (54%) female) admitted by our acute medicine team over the 4-month period, any delirium occurred in 95 patients (31%) (67 with prevalent delirium of whom 17 had recurrent episodes and 28 with incident delirium). Rates of missing data for parameters required for score completion were generally low (functional dependency, *n* = 14; SIRS, *n* = 3; infection, *n* = 7; age, *n* = 0; visual impairment, *n* = 14; dehydration, *n* = 18) except for cognitive test (*n* = 79, no reason documented; *n* = 12, too unwell; *n* = 3, dysphasic; *n* = 1, no English).

AUCs for the different risk scores for any and incident delirium are shown in Table 2 and Supplementary data, Appendix Figure 1, available in *Age and Ageing* online. AUCs ranged from 0.69 to 0.76 for any delirium and 0.73 to 0.83 for incident delirium with no major difference after imputation of missing cognitive data (Table 2). No score was clearly superior (Supplementary data, Appendix Table 1, available in *Age and Ageing* online), and all scores performed better than expected on the basis of chance. Scores predicted any delirium even when originally developed for incident delirium and vice versa. Comparing the original published internal validations of the existing risk scores with the external validations in our cohort (Table 2) showed similar AUC values (Inouye *et al.*, internal validation = 0.66, 0.55–0.77 versus external validation = 0.73, 0.62–0.84; Martinez *et al.*, internal validation = 0.85, 0.80–0.88 versus external validation = 0.69, 0.62–0.76; Isfandiaty *et al.*, internal validation = 0.82, 0.78–0.88 versus external validation = 0.83, 0.74–0.91, Douglas *et al.*, internal validation = 0.69, 0.54–0.83 versus external validation = 0.78, 0.68–0.88), the score (Martinez *et al.*) with greatest discrepancy being originally derived from retrospective chart reviews and requiring major modification.

**Table 2.** AUC for delirium risk scores in acute medicine: original internal validations and validations in our cohort

AUC, 95% CI, delirium					
Score	Internal validation		External validation in our cohort		
	Any	Incident	Any	Incident	Prevalent
Inouye *et al*. [5]		0.66, 0.55–0.77	0.73, 0.66–0.80 *n* = 205 0.74, 0.68–0.80^a^ *n* = 290	0.73, 0.62–0.84 *n* = 149 0.70, 0.60–0.81^a^ *n* = 225	0.70, 0.62–0.72 *n* = 205 0.73, 0.66–0.80^a^ *n* = 290
Martinez *et al*. [6]	0.85, 0.80–0.88		0.69, 0.62–0.76 *n* = 207 0.71, 0.65–0.78^a^ *n* = 294	0.78, 0.68–0.88 *n* = 150 0.75, 0.65–0.84^a^ *n* = 227	0.62, 0.53–0.70 *n* = 207 0.67, 0.60–0.74^a^ *n* = 294
Isfandiaty *et al.* [7]		0.82, 0.78–0.88	0.76, 0.70–0.83 *n* = 205 0.77, 0.71–0.82^a^ *n* = 292	0.83, 0.74–0.91 *n* = 150 0.77, 0.67–0.86^a^ *n* = 227	0.69, 0.61–0.77 *n* = 205 0.73, 0.60–0.80 *n* = 292
Douglas *et al.* [8]		0.69, 0.54–0.83	0.74, 0.67–0.81 *n* = 206 0.75, 0.69–0.81^a^ *n* = 305	0.78, 0.68–0.88 *n* = 150 0.73, 0.63–0.83^a^ *n* = 239	0.68, 0.60–0.76 *n* = 206 0.73, 0.66–0.80^a^ *n* = 305

^a^AUC obtained after imputation of missing cognitive data, missing data assumed normal. In external validations, *n* refers to the number in the sample to which the scores were applied

Table 3 shows the sensitivities, specificities, positive and negative predictive values for all the risk scores for any and incident delirium. In age-stratified analyses, increasing number of risk factors were associated with increased delirium risk irrespective of age, but older age was associated with both a higher prevalence of multiple factors and greater susceptibility (Supplementary data, Appendix Table 2 and Appendix Figure 2, available in *Age and Ageing* online).

**Table 3.** Sensitivity, specificity, positive and negative predictive values for any and incident delirium for each of the four delirium risk scores

Score		Sensitivity	Specificity	ppv	npv
Any delirium					
Inouye *et al.*	1	0.91	0.34	0.45	0.86
	2	0.57	0.80	0.64	0.76
	3	0.17	0.96	0.72	0.66
	4	0.01	1.00	1.00	0.63
Martinez *et al.*	1	0.90	0.36	0.46	0.85
	2	0.62	0.68	0.54	0.75
	3	0.27	0.88	0.58	0.67
Isfandiaty *et al.*	1	0.96	0.34	0.46	0.94
	2	0.89	0.43	0.48	0.87
	3	0.82	0.55	0.52	0.84
	4	0.74	0.71	0.60	0.82
	5	0.49	0.77	0.55	0.72
	6	0.32	0.95	0.77	0.70
	7	0.14	0.99	0.92	0.66
Douglas *et al.*	1	0.95	0.18	0.41	0.85
	2	0.88	0.50	0.51	0.88
	3	0.70	0.66	0.55	0.79
	4	0.27	0.93	0.70	0.68
Incident delirium					
Inouye *et al.*	1	0.95	0.34	0.19	0.98
	2	0.52	0.80	0.31	0.91
	3	0.14	0.96	0.38	0.87
Martinez *et al.*	1	0.95	0.36	0.19	0.98
	2	0.81	0.68	0.29	0.96
	3	0.38	0.88	0.35	0.90
Isfandiaty *et al.*	1	1.00	0.34	0.20	1.00
	2	0.95	0.43	0.21	0.98
	3	0.90	0.55	0.25	0.97
	4	0.81	0.71	0.31	0.96
	5	0.57	0.77	0.29	0.92
	6	0.48	0.95	0.59	0.92
	7	0.19	0.99	0.80	0.88
Douglas *et al.*	1	0.95	0.18	0.16	0.96
	2	0.95	0.50	0.24	0.98
	3	0.76	0.66	0.27	0.94
	4	0.33	0.93	0.44	0.90

## Discussion

Delirium risk scores, using data acquired by the clinical team in the course of routine assessment and including a short cognitive test, reliably risk stratified patients for both any and incident delirium.

Our inclusive cohort of all patients aged ≥65 had delirium rates consistent with reported prevalences of 18–35% and incidences of 11–14% for acute medicine cohorts of ≥100 subjects [1, 22]. Rates are also similar to recent UK studies with different methodologies: 37% in consecutive acute medicine admissions aged ≥75 in Cardiff [23] and 27% in consecutive emergency acute geriatric, medicine and trauma orthopaedic admissions (aged ≥70) in Nottingham [24].

While it is probable that much poor outcome associated with delirium is not preventable, better recognition will facilitate optimal care and targeting of staffing resources to prevent avoidable deterioration, complications and deaths in this vulnerable group [1, 26–28]. Our data suggest that since all scores identified both any and incident delirium, such ‘risk’ scores can help in recognition/screening of delirium as well as in prediction of future risk and will help staff to focus on vulnerable groups. This is of particular clinical utility since multicomponent interventions for example, maintenance of normal sleep wake cycles and daily mobilisation, attention to nutrition and hydration, apply to both treatment and prevention of delirium [1].

We found that scores were robust to adaptation for use with data from routine clinical assessment suggesting that, although delirium is multifactorial, most risk is conferred by a few consistent factors. All adapted risk models included score below cut-off on a short cognitive test and it is likely that this carries significant weight through helping recognition/diagnosis of prevalent delirium and identification of cognitive impairment (pre-existing undiagnosed dementia [29] or subsyndromal delirium) in non-delirious patients who are therefore at high risk of incident delirium. However, it should be noted that significant numbers of older patients are untestable at the point of admission to hospital [30], and thus, risk scores incorporating a cognitive test cannot be applied to this group. The higher rates of delirium seen in older patients resulted from greater prevalence of multiple risk factors and also increased susceptibility: for a given number of risk factors, older patients had more delirium.

AUCs were around 0.7–0.8 for all scores, probably because of the inclusion of broadly similar risk factors, and all scores performed better than chance for both any and incident delirium. Our findings are in contrast to a study validating risk scores in a post-operative population in which AUCs were lower, varying between 0.50 and 0.66 [19]. However, in this study, the mean age of the patients was relatively young, the incidence of delirium was low and most patients were undergoing elective surgery. For AUCs in the range of 0.7–0.8 as found in our study, high sensitivity comes at the cost of specificity and vice versa, i.e. there will be significant numbers of false positives and negatives and the reliability of the scores is far from perfect. However, in the context of widespread under-recognition of patients at risk of delirium [1], risk scores would appear good enough to facilitate targeting of multicomponent interventions in high-risk groups.

Strengths of our study include the prospective inclusive cohort design, regular consultant review facilitating delirium diagnosis and pragmatic use of factors available to the medical team as part of routine clinical care. We were thus able to externally validate and compare clinically applicable delirium risk scores on a representative cohort as recommended in the literature [2–4].There are some limitations to our study. First, we did not examine inter-observer reproducibility of delirium diagnosis. However, the diagnosis of delirium was made by experienced physicians/geriatricians. Second, since we performed the study in the course of routine care, diagnosis was not blinded to the patients' clinical characteristics and thus there is the possibility of bias. However, the fact that our observed delirium rate was similar to that reported in other studies suggests that there was no significant over-diagnosis. Third, many patients did not have cognitive testing completed without a documented reason but may have been testable, and this might have impacted on measured AUC values.

In conclusion, our findings have implications for clinical practice. Risk stratification of patients in routine practice can be achieved with simple and feasible delirium risk scores which will facilitate both recognition and prevention of delirium and help target multicomponent intervention. Such risk scores will also enable estimation of delirium rates by case-mix in the general hospital. Finally, our study will aid sample size calculation and selection of high-risk patients for future clinical trials.

**Key points**

- Delirium risk scores are reliable in identifying high-risk patients in acute medicine.
- Delirium risk scores are robust to simplification.
- Delirium risk scores are feasible for use in routine clinical practice.

## Supplementary data

Supplementary data mentioned in the text are available to subscribers in *Age and Ageing* online.

## Authors' contributions

S.T.P. conceived and designed the study, collected data and directed analyses, and wrote the paper. N.L. collected data and performed literature searches. S.C.S. and E.C. collected data. Z.M. designed and performed statistical analyses. P.M.R. helped design analyses and advised on risk score application and co-wrote the manuscript.

## Conflicts of interest

None declared.

## Funding

S.T.P. is supported by the NIHR Oxford Biomedical Research Centre. P.M.R. is an NIHR Senior investigator and a Wellcome Trust Senior Investigator. There was no specific funding for this study, and the sponsors had no role in the study design or analyses.

## Supplementary Material

Supplementary Data
