# Supplementary material for: Delirium risk stratification in consecutive unselected admissions to acute medicine: validation of externally derived risk scores
Source: Age Ageing. 2016 Jan 13;45(1):60–5. doi: 10.1093/ageing/afv177 (PMC4711661; doi:10.1093/ageing/afv177)
Supplement: Supplementary Data [file supp_45_1_60__index.html]

Supplementary Data 

# Delirium risk stratification in consecutive unselected admissions to acute medicine: validation of externally derived risk scores

## Supplementary Data

Supplementary Data

- Supplementary Data - Docx file
