# Supplementary material for: Delirium risk stratification in consecutive unselected admissions to acute medicine: validation of externally derived risk scores
Source: Age Ageing. 2016 Jan 13;45(1):60–5. doi: 10.1093/ageing/afv177 (PMC4711661; doi:10.1093/ageing/afv177)

**SUPPLMENTARY DATA**

**Delirium risk stratification in consecutive unselected admissions to acute medicine: validation of externally derived risk scores**

Sarah T. Pendlebury FRCP DPhil, ^1,2,3^ Nicola Lovett BM BCh, ^2,3^ Sarah C. Smith FRCP, ^2^ Emily Cornish,^2^ Ziyah Mehta DPhil^3^ and Peter M. Rothwell FMedSci.^1,3^

^1^NIHR Oxford Biomedical Research Centre, John Radcliffe Hospital, Oxford, UK

^2^Departments of General (Internal) Medicine and Geratology, John Radcliffe hospital, Oxford, UK

^3^Stroke Prevention Research Unit, Nuffield Department of Clinical Neurosciences, John Radcliffe Hospital and the University of Oxford

Abbreviated Title: Delirium risk stratification in acute medicine

Key words: delirium, prediction, risk stratification, risk scores, acute medicine

Correspondence to:

Prof Sarah Pendlebury

Stroke Prevention Research Unit

Level 6 West Wing

John Radcliffe Hospital

Oxford OX3 9DU

Tel: +44 1865 231603 Fax: +44 1865 234639

Email: [sarah.pendlebury@ndcn.ox.ac.uk](mailto:sarah.pendlebury@ndcn.ox.ac.uk)

Appendix Table 1. Z test for significance of difference between AUC scores for any and incident delirium, all scores tested pairwise against the Inouye score

|  |  |  |
| --- | --- | --- |
|  |  |  |
| Risk Score | AUC (95% CI) | p vs Inouye score |
|  |  |  |
|  |  |  |
| Martinez (any) | 0.69 (0.62-0.76) | 0.71 |
| Martinez (incident) | 0.74 (0.68-0.88) | 0.73 |
|  |  |  |
| Isfandiaty (any) | 0.76 (0.70-0.83) | 0.70 |
| Isfandiaty (incident) | 0.70 (0.59-0.80) | 0.71 |
|  |  |  |
| Douglas (any) | 0.89 (0.77-0.91) | 0.82 |
| Douglas (incident) | 0.74 (0.68-0.88) | 0.75 |
|  |  |  |

Table 2. Mean and median values for all delirium scores calculated using non-age factors stratified by age and presence of delirium.

|  |  | Score Mean+sd  Median | | | | | | | |
| --- | --- | --- | --- | --- | --- | --- | --- | --- | --- |
|  |  | Inouye | | Martinez | | Isfandiaty | | Douglas | |
|  |  | yes | no | yes | no | yes | no | yes | no |
| Any delirium | Age<80 | 1.3+1.0 | 0.7+0.7 | 1.0+0.7 | 0.5+0.7 | 3.6+2.0 | 1.9+1.9 | 1.8+1.1 | 0.9+1 |
|  |  | 1.0 | 1.0 | 1.0 | 0.0 | 4.0 | 2.0 | 2.0 | 1.0 |
|  | Age>80 | 1.8+0.9 | 1.0+0.8 | 1.3+0.7 | 0.8+0.8 | 4.6+1.9 | 2.6+2.2 | 2.1+0.9 | 1.2+1.1 |
|  |  | 2.0 | 1.0 | 1.0 | 1.0 | 5.0 | 3.0 | 2.0 | 2.0 |
|  |  |  |  |  |  |  |  |  |  |
| Incident delirium | Age<80 | 0.8+0.5 | 0.7+0.7 | 1.0+0.8 | 0.7+0.5 | 3.3+1.7 | 1.8+1.9 | 1.5+1.0 | 0.9+1.0 |
|  |  | 1.0 | 1.0 | 1.0 | 0.0 | 3.5 | 2.0 | 2.0 | 1.0 |
|  | Age>80 | 1.8+0.7 | 1.0+0.8 | 1.6+0.6 | 0.8+0.8 | 5.3+1.5 | 2.6+2.2 | 2.4+0.6 | 1.2+1.1 |
|  |  | 2.0 | 1.0 | 2.0 | 1.0 | 6.0 | 3.0 | 2.0 | 2.0 |
|  |  |  |  |  |  |  |  |  |  |
| Prevalent delirium | Age<80 | 1.4+1.1 | 0.7+0.7 | 0.9+0.7 | 0.5+0.7 | 3.6+2.1 | 1.9+1.9 | 1.9+1.2 | 0.9+1.0 |
|  |  | 1.5 | 1.0 | 1.0 | 0.0 | 4.0 | 2.0 | 2.0 | 1.0 |
|  | Age>80 | 1.8+1.0 | 1.1+0.9 | 1.2+0.8 | 0.8+0.9 | 4.3+1.9 | 3.0+2.3 | 2.0+0.9 | 1.4+1.1 |
|  |  | 2.0 | 1.0 | 1.0 | 1.0 | 4.0 | 3.0 | 2.0 | 2.0 |

Appendix Figure 1. AUCs for existing delirium risk scores for any (top), incident (middle) and prevalent (bottom) delirium.


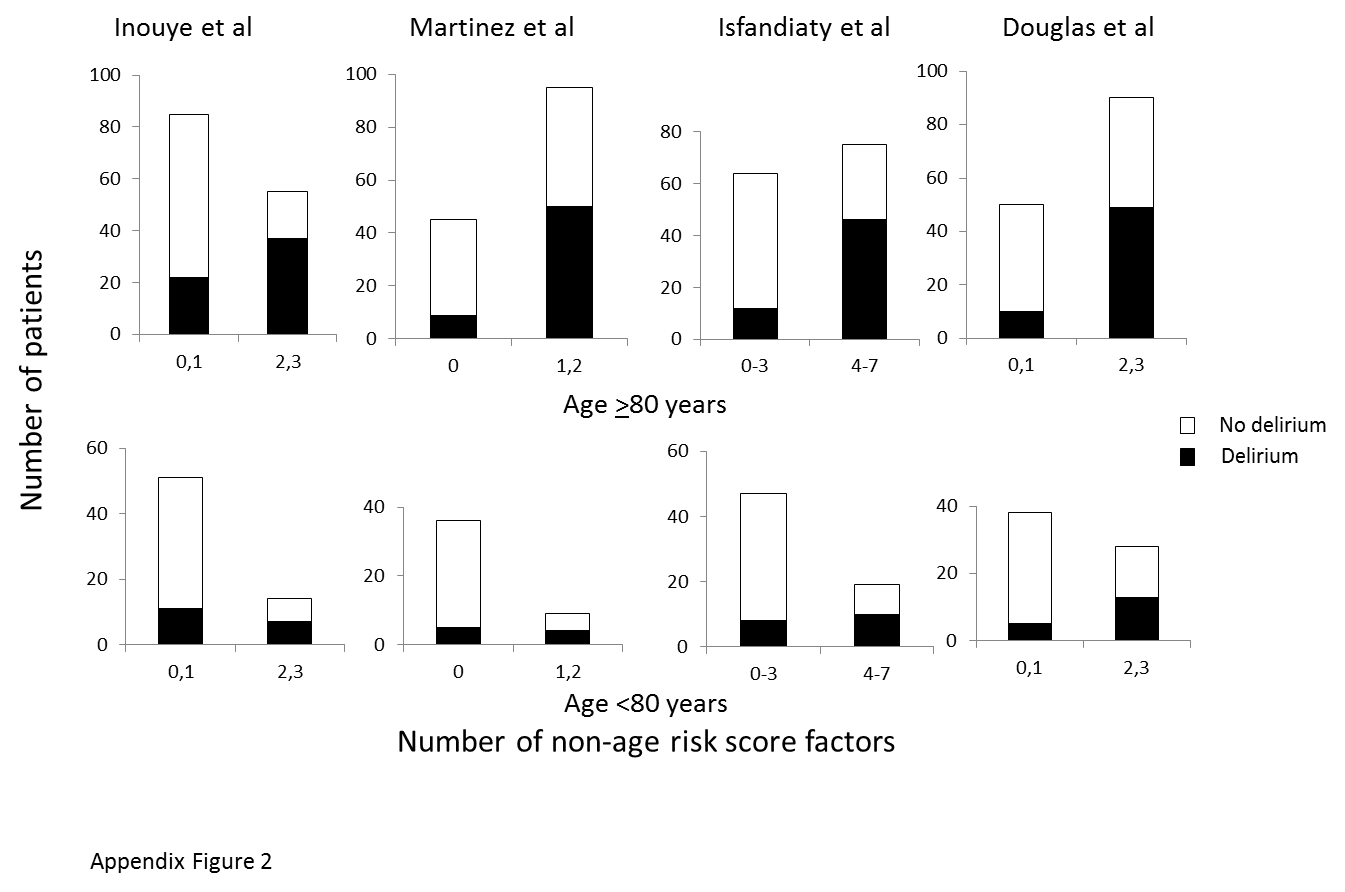

Supplement: Supplementary Data [file supp_afv177_afv177supp.docx]
